# Perceived Pain in Athletes: A Comparison between Endurance Runners and Powerlifters through a Cold Experimental Stimulation and Two Sessions of Various Physical Activation

**DOI:** 10.3390/sports10120211

**Published:** 2022-12-19

**Authors:** Pierluigi Diotaiuti, Angelo Rodio, Stefano Corrado, Stefania Mancone, Fernando Bellizzi, Thais Cristina Siqueira, Alexandro Andrade

**Affiliations:** 1 Department of Human Sciences, Society and Health, University of Cassino and Southern Lazio, 03043 Cassino, Italy; 2 Health and Sports Science Center, Department of Physical Education, CEFID, Santa Catarina State University, Florianópolis 88035-901, Brazil

**Keywords:** perceived pain, endurance runners, powerlifters, cold pressor test, aerobic training, strength training, blood pressure

## Abstract

Few studies in the literature have illustrated cold hypoalgesia induced by strength training. Objectives of this contribution were to compare the ratings of perceived pain in endurance running (n = 22) and powerlifting (n = 22) male athletes and controls (n = 22) at baseline and after two bouts of 40 min aerobic/strength training respectively, using the Cold Pressor Test (CPT) and simultaneously monitoring changes in blood pressure (BP), heart rate (HR), and body temperature. A two-way repeated measures ANOVA was conducted to examine the effects of training sessions in endurance runners vs. powerlifting athletes vs. controls on the intensity of perceived pain at CPT. A statistically significant two-way interaction between the group and training resulted in *p* < 0.001, ηp^2^ = 0.513. A simple main effects analysis showed that as the participants went through the strength training session, pain perception at CPT was significantly lower in powerlifters compared to runners and controls. Considering the physiological parameters, powerlifters reported significantly higher values of BP and HR. This difference was present at baseline but after training as well, and before and after CPT, despite a slight hypotensive effect. The differences reported after CPT at baseline, but very significantly after the strength activation session in the powerlifters, provide interesting insights into the hypoalgesic effect of high-intensity strength training.

## 1. Introduction

Exercise-induced hypoalgesia (EIH) is characterized by a decrease in sensitivity to painful stimuli, with variable duration, lasting up to 30 min after a single bout of exercise. According to Rice et al. [1], the precise physiological mechanisms underlying exercise-induced hypoalgesia are currently unknown. Analgesia following exercise appears to be most consistent when the exercise stimulus involves exercise performed at higher intensities (i.e., >70% of maximal aerobic capacity), and animal research suggests that properties of the exercise stressor are important in determining which analgesic system is activated during exercise [2]. Hypoalgesia after aerobic exercises (e.g., cycling or running), dynamic resistance exercises (e.g., circuit training), and isometric exercises (e.g., a wall squat) often produce an increase in pressure pain thresholds [3,4]. It has been pointed out that exercise intensity quite consistently affects the EIH response after aerobic exercise [5,6,7] and also after isometric exercises [8,9,10,11]. Some studies have demonstrated that exercise which is presumed to be more painful (higher intensity submaximal isometric exercise) produces a greater EIH response than exercises that are presumed to be less painful (lower intensity submaximal isometric) [8]. However, others have found pain ratings to be unrelated to EIH response [12]. Considering the few studies of EIH that assess the effects on cold pain, the majority examines aerobic exercise [13,14,15], some focus on isometric exercises [11], while there appear to be few studies in the literature that have illustrated cold hypoalgesia induced by strength training. These include Samuelly-Leichtag et al. [16], who revealed that high-intensity exercise, even for a short duration, induced a hypoalgesic effect for pressure, heat, and cold modalities.

The analgesic effect of aerobic and anaerobic exercise is usually associated with an increase in the peripheral concentration of Beta-endorphin and with the activation of (supra)spinal nociceptive inhibitory mechanisms orchestrated by the brain [17]. In the first case (anaerobic), hypoalgesia occurs when the anaerobic threshold is exceeded as a result of short-duration exercise with a progressive increase in intensity [16,18]; in the second case (aerobic), it occurs after about an hour of continuous exercise with a constant condition of production and elimination of lactate [19,20]. As already reported in Vaegter and Jones [4]., a single session of exercise has repeatedly been observed to reduce pain sensitivity in pain-free individuals. Scheef et al. [21] suggested that running exercise reduced the pain-induced activation in the periaqueductal gray, a key area in descending pain inhibition which, in turn, was associated with lower pain unpleasantness ratings to thermal stimuli. Although athletes, in general, seem less responsive to noxious stimuli than non-athletes, the type of sport differentially affects pain perception. A recent study comparing aerobic and anaerobic hypoalgesia in athletes was carried out by Assa et al. [22]. They compared endurance athletes, who perform continuous intense activity for prolonged periods, with strength athletes who perform short-duration exercises at very high intensity, reporting significant differences related to pain sensitivity and inhibition of the nociceptive stimulus: athletes in endurance sports showed greater pain inhibition, whereas strength athletes showed reduced pain sensitivity. They distinguished between two distinct processes: inhibition as an efficient top-down inhibitory control mechanism that results in conditioned pain modulation (CPM) and which in endurance athletes is closely related to motivational components, such as the will to resist pain, and reduced pain sensitivity, which would correspond in powerlifters primarily to a different distribution/density of skin nociceptors, attributable to the muscle hypertrophy that accompanies this type of training. They, therefore, with this work, emphasized the contribution of training specificity on pain perception.

Very few studies have examined the relationship between resistance training and pain modulation. However, a study carried out by Koltyn and Arbogast [23] concluded that a single bout of resistance exercise can achieve a hypoalgesic response. The resistance exercise consisted of 45 min of lifting 3 sets of 10 reps at 75% of 1-Repetition Maximum (1 RM), which included bench presses, leg presses, pull-downs, and arm extensions. A further study performed by Vaegter et al. [24] had their subjects perform two isometric contractions of dominant biceps brachii and quadriceps at 30% and 60% Maximum Voluntary Contraction (MVC). They concluded that high-intensity isometric contraction by biceps brachii and quadriceps produced a larger local EIH compared to low-intensity contraction.

From the literature review, we could see that there have been numerous studies on how long-distance runners perceive and cope with pain, while no research has been performed with athletes practicing disciplines of short-duration and maximal force development such as jumping, wrestling, and powerlifting [25]. Other studies have previously applied CPT as pain evoking stimulus to evaluate the analgesic effect of pharmacological and psychological treatments [26,27]. Our aims were to measure differences in perceived pain intensity between the groups of athletes both in the baseline condition (i.e., after a 120 s immersion test of the non-dominant hand in the ice-water container) and with a second measurement after stimulating the athletes with two 40 min training sessions, conducted on a treadmill and with pyramid strength training, respectively.

Only one prior study has examined the analgesic effect of aerobic training in runners using multiple pain stimuli (including CPT) and was conducted by Janal et al. [13]. They showed that no significant analgesic response was found for the cold pressor test. A similar result is reported by Ruble et al. [14], where, however, the participants were healthy volunteers and not athletes. Although there is no specific evidence for the category of powerlifters, we found useful what was indicated regarding the hypoalgesic effects of a resistance exercise session by Koltyn and Arbogast [23].

Following the aforementioned literature, ratings of perceived pain intensity at CPT were therefore hypothesized to be in any case lower in the athletes compared with controls at baseline and significantly even lower than the controls when the athletes later performed the training session corresponding to their discipline (aerobic for runners and anaerobic for powerlifters). We, therefore, hypothesized that the training session corresponding to the type of physical work usually performed by the athlete could produce a rapid activation of the usual physiological adaptation processes of the athlete’s body, also involving the sphere of modulation of perceived pain.

## 2. Materials and Methods

### 2.1. Participants

The population was characterized by university student athletes who attend the University of Cassino and Southern Lazio. Given the ongoing collaboration agreement between the university where this study took place and FIPE (Italian Weightlifting Federation), we chose to involve powerlifters among the maximal force development disciplines. We, therefore, believed that this comparison (running vs. powerlifting athletes) could contribute to increasing knowledge of the mechanisms that lead to pain inhibition and different pain sensitivity in sports. Therefore, in our study, we have chosen the line of comparison of pain sensitivity between athletes from different disciplines [22,25], and among the university student athletes, we involved a group of endurance runners, a group of competitive powerlifters, and a control group of healthy non-athlete students. The differences in pain perception have been assessed through the pain induction achieved by the Cold Pressor Test (CPT). A statistical power analysis was performed for sample size estimation. The effect size (ES) in this study was set to 0.30, considered to be medium, using Cohen’s criteria. Through the G*Power 3.1 software (Düsseldorf, Germany), the minimum number of participants needed with this effect size and appropriate to perform an intergroup comparison was preliminarily set at 33. Through an open invitation sent out to the entire study population and supported by the University Sports Center, the sample was gathered in a non-probabilistic manner.

The students were made aware of their involvement in a lab experiment to gauge individual sensitivity to cold. Inclusion criteria were: (1) age range of 18–28 years, and (2) competitive athletes of endurance running or powerlifting at regional level at least, who have trained regularly over the last three years. Exclusion criteria were: (1) inability to understand and follow instructions in verbal and written Italian, (2) any health conditions potentially causing sensory deficits, such as diabetes mellitus or neurological disorders, (3) any history of chemotherapy, (4) the current assumption of medication that can affect sensation, and (5) a current pregnancy.

Students who were interested in participating were encouraged to sign up by clicking a special link in the announcement. Once the link was opened, it allowed students to enter their contact information as well as their gender and sport preference (endurance running; powerlifting). The researchers then planned and informed the students of the precise dates and times they would be required to report to the lab. Regarding the study’s design, since it compared how endurance runners and powerlifters perceived pain on the CPT test, the list of those booked for the study was divided so as to randomly allocate the same number of participants to both groups.

A total of 51 student-athletes, all of whom were male, expressed a desire to participate in the study; however, 7 of them, who had been placed on the list after random group assignment, were unable to take the test. As a result, 44 athletes participated in the final count. As control subjects, 22 healthy non-athlete students engaged in general fitness activities (gym, amateur sports) belonging to the same age group (18–28) were also recruited on a voluntary basis, and the same exclusion criteria as above were applied. Therefore, our final sample size of 66 participants should be considered more than sufficient to achieve the main objective of this study. No drop-outs were noted because everyone who began the test finished it on time.

Prior to the testing session, selected participants were instructed to abstain from caffeine, alcohol, and any medications that might make them drowsy or analgesic for 24 h. Before data collection started, the procedure was explained, and written informed consent was obtained. The study was carried out in accordance with the Helsinki Declaration guidelines and was approved by the Institutional Review Board of the University of Cassino and Southern Lazio (IRB_SUSS_08:18/04/19). Table 1 shows the baseline anthropometric data of the participants.

### 2.2. Procedures

**Figure 1:**
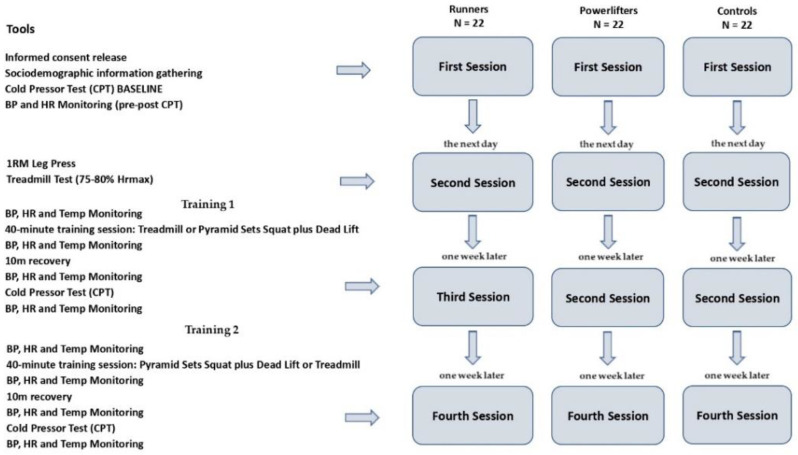
Method flowchart.

Each of the participants—all volunteers—was called to the lab for morning sessions (in the 9 a.m.–12 p.m. time slot). Following Figure 1 shows the method flowchart employed.

All athletes were invited to the laboratory four times. The first time, they (a) obtained information about the study from the researchers, (b) gave informed consent to take part, and were told about the safety of the scientific and aggregate use of the data they gave, as required by the Declaration of Helsinki, (c) filled out a preliminary questionnaire to collect demographic data, and (d) took part in the CPT session.

The CPT was chosen as a method for inducing and measuring changes in pain perception. Participants could stop the test whenever they thought the pain was too much to handle. Before the execution of the CPT, the protocol included measurement of heart rate and BP (systolic and diastolic), then the participants were asked to immerse their non-dominant hand for two minutes in a basin containing three liters of room temperature water, both to accustom the hand to low temperatures and to make the participants’ basal temperatures homogeneous before the test. The Polar heart rate device M460HR (Kempele, Finland) was used to measure HR frequency, while the Omron sphygmomanometer M2 (Osaka, Japan) was used for the detection of BP. The participant was then asked to place his/her non-dominant hand and wrist in 8 °C water in a 13 L plexiglass container connected to a circulating water bath (Termocriostat CF40-HE, Julabo GmbH, Seelbach, Germany) and maintain it there as long as he/she could or preferably until the maximum time limit of 120 s was reached. Although healthy adults are typically given a maximum immersion time of three to five minutes [28,29,30], the test limit of two minutes was selected in order to completely reduce the risk of tissue injury [31].

The perception of pain intensity was evaluated by a *Visual Analog Scale* [32,33], scoring on an 11-interval numerical graduation scale (0 = no perceived pain, 10 = maximum perceived pain). In our case, the subject was asked to immerse their non-dominant hand and indicate the progression of pain perception from the beginning to the end of the test (120 s) with their index finger on the other hand. When the discomfort became unbearable, all participants were given the option to withdraw their hands and stop the test. In case of withdrawal, the participant was given the highest score on the final pain perception record. A portable camera (Camcorder FHD 1080P, Panasonic, Kadoma, Japan) placed on support behind the subject allowed the recording of the values of the progression of perception indicated on paper on a scale of 1–10. For the purposes of the test, the intensity of pain felt by the athlete at the end of the 120 s test was recorded. The stability of the water temperature in the tank was also controlled with the help of an internal LCD thermometer with suction cup (Ueetek, Shenzhen, China) and using an external infrared thermometer device (IR, KKmoon, Guangdong, China). A maximum temperature fluctuation of 3 °C was allowed in the tank. At the end of 120 s of cold-water immersion, the participant’s heart rate and BP were measured again.

The second session in the laboratory took place the following day. The objective was to determine for all (athletes and controls) the physical workload that would be performed in an additional session in order to assess the change in BP and any concomitant effect on CPT-induced pain perception. It was decided to have a 40 min aerobic session on treadmill at moderate intensity with 75–80% maximum heart rate (HRmax) and a strength training session using pyramid sets of increasing weight (10-8-6-4 repetitions) with 240 s recovery on the Squat and Dead Lift, for a total duration of 40 min. In the latter case, exercises directly involving the upper limbs (such as the Bench Press) were intentionally excluded in order to avoid confounded findings related to a local hypoalgesia at the hand that would then be dipped into the cold box. Therefore, in this laboratory session, athletes and controls were subjected to the 1 RM at the Leg Press to assess the maximum load from which to derive the weight distribution for the pyramid series and the Treadmill Test to identify the individual maximum heart rate from which to derive the established rate percentage (75–80% HRmax) [34,35]. Based on these data, individual physical work programs were prepared for the participants to perform in the next two sessions. The sessions were carried out with Technogym equipment (Cesena, Italy) in the university gym. Athletes’ weight and height were measured through the mobile measurement device SECA Mod. 284 (Hamburg, Germany).

The third session in the laboratory took place one week later. Participants underwent a specific 40 min training session in the university gymnasium before performing the CPT again in the laboratory (located right next to the gymnasium). In order to comparatively evaluate the hypoalgesic effect of the aerobic and strength training sessions, each participant (both athlete and control) performed the two physical pieces of training in two separate sessions spaced one week apart. In order to mitigate any habituation effect, the succession of the two types of training was counterbalanced within the three groups. Thus, in this third session, half of each group was randomly assigned the aerobic session, while the remaining halves were assigned strength training.

In the fourth and final session (one week later), for each participant, the type of physical training was reversed so that those who had performed aerobic training in the third session performed strength training in the fourth session (and vice versa).

As established, the aerobic training session consisted of a short warm-up on the exercise bike (five minutes) and forty minutes on treadmill at moderate intensity of 75–80% HRmax, while the strength training session, after the same short warm-up on the exercise bike (5 min), was composed of pyramid sets of increasing weight (10-8-6-4 repetitions) with 240 s recovery on the Squat and Dead Lift, for a total duration of 40 min. At the end of the training sessions, participants went to the laboratory to perform the cold pressor test.

Compared to the procedure performed at baseline during the first session, in addition to BP and heart rate measurements, body temperature was measured four times on forehead: before training, at the end of training, before CPT, and at the end of CPT.

### 2.3. Statistical Analysis

Statistical analyses were performed using the package SPSS (IBM, Chicago, IL, USA) version 26 and JAMOVI version 2.2.5 for data presentation through violin plots. The verification of the assumptions of univariate normality was conducted using the procedure for the standardization of the variables by inspection of a boxplot and using Shapiro–Wilk’s normality test and Levene’s test of homogeneity of variance. A Welch’s F-test was run to evaluate differences in perceived pain intensity between athletes and controls at baseline, and a Games–Howell post hoc test to assess pairwise differences between group means.

In order to compare the perceived pain in the groups of athletes before and after the training session, a two-way repeated measures ANOVA was run with two independent variables (group, training) and one dependent variable (pain perception at CPT). The primary purpose is to understand if there is an interaction between these two factors on the dependent variable, whereas, to compare the physiological values (BP, HR, BT) in the groups of athletes before and after the training sessions, three-way repeated measures ANOVAs were run. As the number of participants in the groups was balanced, in order to determine the interaction between the variables, Pillai’s criterion was used instead of Wilks’ Lambda as it is more robust to unequal covariance matrices [36]. Following Cohen (1998) [37], partial Eta squared (ηp^2^) was the measure used to assess effect size (0.01 = small, 0.06 = medium, 0.13 = large).

The level of significance was set at *p* < 0.05, while for the testing of multiple univariate interaction effects, a Bonferroni adjustment was introduced by dividing the declared level of statistical significance by the number of dependent variables: *p* < 0.025 (i.e., *p* < 0.05 ÷ 2). The follow-up investigation proceeded with the computation of simple effects tests in order to reveal the degree to which one factor was differentially effective at each level of the second factor.

## 3. Results

The score of perceived pain intensity was statistically significantly different between the groups, Welch’s F = 24.38, *p* < 0.001. Perceived pain intensity increased from the powerlifters (M = 6.45, SD = 2.11) to runners (M = 7.73, SD = 1.96) and to controls (M = 9.36, SD = 0.58), in that order.

A Games–Howell post hoc analysis revealed that the mean increase from runners to controls (1.64, SE = 0.43, 95% CI [0.55, 2.72]) was statistically significant (*p* = 0.003), as well as the increase from powerlifters to controls (2.91, SE = 0.47, 95% CI [1.74, 4.07], *p* = 0.000); instead, the mean increase from powerlifters to runners was not significant (1.27, SE = 0.61, 95% CI [−0.22, 2.76], *p* = 0.107).

**Figure 2:**
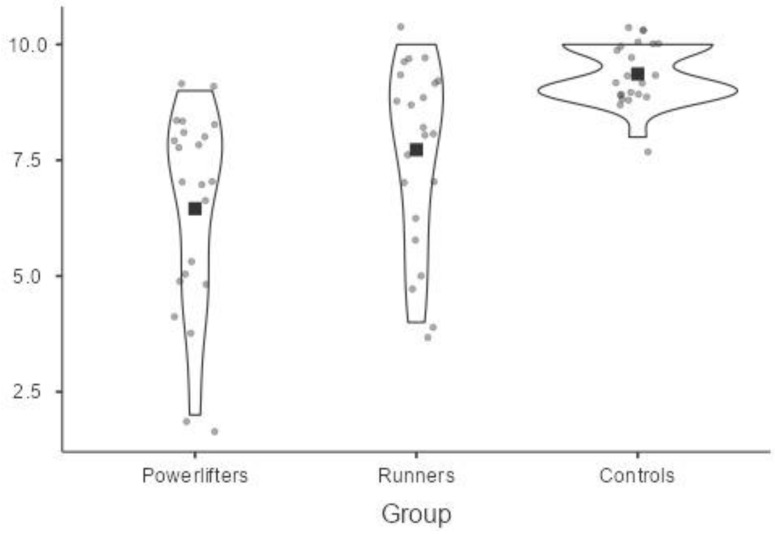
Perceived pain intensity at CPT among athletes and controls.

The following Figure 2 shows through violin plots the difference in perceived pain intensity at CPT between the athletes and the controls at baseline. On each side of the line is a kernel density estimation to show the distribution shape of the data. Wider sections of the violin plot represent a higher probability that members of the population will take on the given value; the skinnier sections represent a lower probability.

Table 2 below shows the BP (diastolic and systolic) and HR readings collected in all participants at baseline, before and immediately after the conclusion of CPT. A comparison of the mean values reported in the bottom row of the table shows that the powerlifters group had higher BP (systolic and diastolic) and HR values than the other two groups both in the baseline measurement and after performing CPT.

The significance of the effect resulted with regard to the group variable (*p* < 0.025; ηp^2^ = 0.622) and the training variable (*p* < 0.025; ηp^2^ = 0.378). There was a statistically significant two-way interaction between the group and training: F(2.757, 57.903) = 22.095 *p* < 0.025, ηp^2^ = 0.513. Therefore, simple main effects were run: as the participants went through the strength training session, pain perception at CPT was statistically significantly different in powerlifters (M = 4.36, SD = 1.92) compared to runners (M = 7.91, SD = 1.69) with a mean difference of −3.54, 95% CI [−4.59, −2.50], and to controls (M = 9.41, SD = 0.59) with a mean difference of −5.05, 95% CI [−5.89, −4.20], F(2, 42) = 69.978, *p* < 0025, partial η^2^ = 0.769. As the participants went through the aerobic training session, pain perception at CPT was statistically significantly different in powerlifters (M = 7.09, SD = 1.02) compared to controls (M = 9.45, SD = 0.51) with a mean difference of −2.36, 95% CI [−2.81, −1.92], but not to runners (M = 7.36, SD = 2.24) with a mean difference of −0.27, 95% CI [−1.44, 0.90], F(1.204, 25.275) = 17.087, *p* < 0025, partial η^2^ = 0.449.

Turning to the simple main effects of training, in powerlifters, the pain perception at CPT was statistically significantly different after the strength training (M = 4.36, SD = 1.92) compared to the pain perception after the aerobic training (M = 7.09, SD = 1.02) with a mean difference of −2.09, 95% CI [−2.99, −1.19], and with respect to the non-training condition (M = 6.45, SD = 2.10) with a mean difference of −2.73, 95% CI [−3.59, −1.86], F(2, 42) = 26.460, *p* < 0.025, partial η^2^ = 0.558.

In runners, the pain perception at CPT was statistically significantly different but >0.025 after the aerobic training (M = 7.36, SD = 2.24) compared to the pain perception after the strength training (M = 7.91, SD = 1.69) with a mean difference of −0.55, 95% CI [−1.05, −0.04], and to the non-training condition (M = 7.73, SD = 1.96) with a mean difference of −0.36, 95% CI [−0.65, −0.07], F(1.481, 31.096) = 3.973, *p* = 0.04, partial η^2^ = 0.159.

In controls, the pain perception at CPT was not statistically significantly different (*p* > 0.05) comparing aerobic training (M = 9.45, SD = 0.51), strength training (M = 9.41, SD = 0.59) with a mean difference of 0.05, 95% CI [−0.33, 0.42], and non-training (M = 9.36, SD = 0.58) with a mean difference of −0.05, 95% CI [−0.37, 0.28], F(2, 42) = 0.137, *p* = 0.872, partial η^2^ = 0.006.

**Figure 3:**
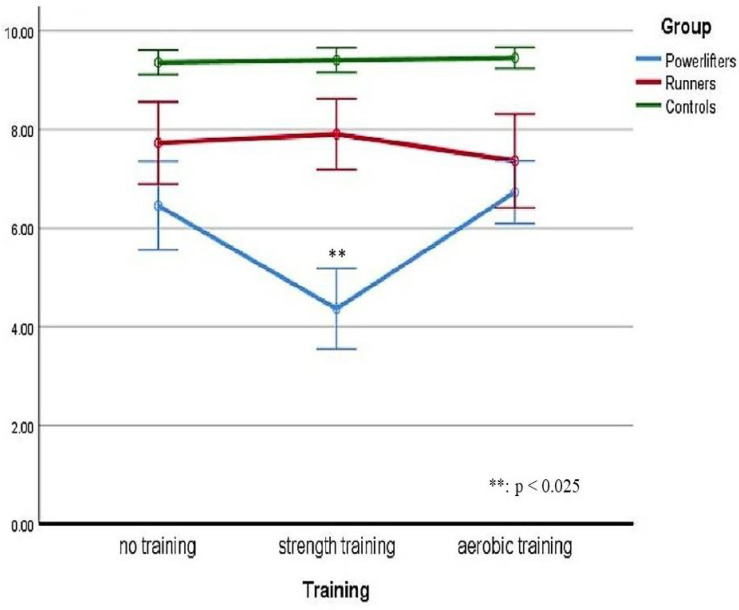
Perceived pain considering both groups and the training factor.

The following Figure 3 shows overall trends in the perceived pain intensity considering both groups belonging and the training factor. As can be noted, the most significant effect is found in the Powerlifters group after they had performed the strength training session.

Regarding the physiological measures considered in the study, in relation to the SBP variable, there was not a statistically significant three-way interaction between group, training, and time, F(1, 21) = 2.594, *p* > 0.001; nor did they turn out to be simple two-way interactions: group*time, F(1, 21) = 0.470, *p* > 0.001; group*training, F(1, 21) = 0.439, *p* > 0.001; time*training, F(1, 21) = 1.743, *p* > 0.001. Related to the DBP variable, there was a statistically significant three-way interaction between group, training, and time, F(1, 21) = 16.320, *p* < 0.005. Two simple two-way interactions were also being reported: group*time, F(1, 21) = 32.881, *p* < 0.001; time*training, F(1, 21) = 14.757, *p* < 0.001, but not group*training, F(1, 21) = 2.037, *p* > 0.001. In relation to the HR variable, there was not a statistically significant three-way interaction between group, training, and time, F(1, 21) = 3.460, *p* > 0.001. However, three simple two-way interactions were recorded: group*time, F(1, 21) = 21.803, *p* < 0.001; group*training, F(1, 21) = 132.877, *p* < 0.001; time*training, F(1, 21) = 62.334, *p* < 0.001. Relating to the variable BT, there was a statistically significant three-way interaction between group, training, and time, F(1, 21) = 15.069, *p* < 0.001. Two simple two-way interactions were also found: group*time, F(1, 21) = 10.911, *p* < 0.025; time*training, F(1, 21) = 9.464, *p* < 0.025; but not group*training, F(1, 21) = 0.467, *p* > 0.001. The simple main effects were then tested.

All physiological values recorded can be consulted in the Tables S1–S6 in Supplementary Materials. Table 3 below shows the overall significant differences between the mean values of the physiological measurements collected in the three groups (powerlifters, runners, and controls) before and after training (both aerobic and strength) and before and after CPT. It can be seen that the group of powerlifters reported significantly higher values of BP and HR. This difference was present at baseline but after training as well, and before and after CPT, despite a slight hypotensive effect recorded, while body temperature measurements showed no significant changes and differences both before and after training and CPT and in the comparison between the groups.

## 4. Discussion

Several studies in the literature have illustrated evidence of the acute effects of experimental manipulation on pain perception, emphasizing that certain conditions can significantly contribute to a reduction in pain perception. Some have performed manipulations based on psychological variables using biofeedback, guided imagery, hypnosis, and distraction techniques [38,39,40,41,42], while others have assessed the incidence of individual variables such as gender, experience, and anthropometric characteristics [43,44,45,46]. Others still have assessed the incidence of different sports and exercise activities [47,48,49,50].

In our study, which is part of this strand of sports research, we aimed to consider differential effects in pain manipulation induced by an intense training session in athletes from two different disciplines and in healthy controls. The results of our study indicate that a single bout of training activates in athletes a significant decrease in perceived pain in the CPT test compared to a baseline measurement, and compared to a group of distance runners, this effect is especially pronounced among powerlifters. Even at baseline, a difference is reported (although not significant), but it is in line with the post-training trend: powerlifters still reported a lower perceived intensity of pain after 120 s of CPT. If we consider the comparison with the control group at baseline, the results are consistent with the aforementioned prior research that had pointed out the general lower sensitivity to pain in athletes compared to healthy controls, regardless of the discipline practiced.

On the other hand, these results, taken together, suggest different conclusions from previous studies that have emphasized the greater analgesic effect of aerobic exercise and purely aerobic sports such as endurance running [14]. An explanation for this evidence could be found, for example, in the study of Bond et al. [51], where it emerged that the group that had followed an aerobic training program had recorded at CPT a particular adaptation response to the situation, consisting of a significant reduction in systolic and diastolic BP levels, a response not present in the control group. A similar result is reported by Gideon et al. [52] and Yoon et al. [53]. Therefore, it could be hypothesized that aerobic practice predisposes to a better adaptive response to stressful situations such as the CPT. Jones et al. [19] have already highlighted how increasing the intensity of aerobic exercise from moderate to vigorous increased pain tolerance in healthy non-athletic individuals. In this regard, more recent studies have reported this progressive adaptation of pain tolerance in endurance runners, emphasizing the positive weight of the psychological attitude oriented to the control of the situation [54,55].

Geva and Defrin [56] reported important findings regarding pain tolerance in athletes of multidisciplinary sports such as triathlon, an extremely intense sport that exposes performers to considerable physical and psychological suffering during training and competition. These athletes showed higher pain tolerance, giving lower pain perception and fear level ratings than the control group. Similar results with triathlon athletes are shown in Gagnon-Dolbec et al. [57] research. This evidence prompts the hypothesis that athletes who compete in difficult long-distance races possess very specific psychological characteristics that enable them to adapt to extremely harsh conditions [58].

Other studies have shown the analgesic effect of anaerobic exercise programs based on acute dynamic resistance [23,47,59,60,61]. As pointed out in the meta-analytic review by Nagle et al. [62], in the few studies that have used acute dynamic resistance, it appears that intermittent exercise, and not just continuous exercise, is capable of producing medium-to-large EIH effects. However, it would still be necessary to deepen the determination of the threshold of dynamic resistance exercise required to produce EIH and whether EIH elicited by dynamic resistance exercise generalizes to other types of pain stimuli (such as cold).

Considering the above, our study can contribute to providing further evidence both through the choice of the sample of powerlifters, trained constantly in the use of explosive, rapid, and intermittent force, directly compared with a sample of resistance runners (instead of healthy controls as performed mostly in other previous studies) and through the use of the measurement of perceived pain intensity through the CPT methodology. A number of studies link the reduction of sensitivity in anaerobic athletes to their body mass and/or musculature, the formers being more hypertrophic. Hypertrophic musculature may affect the density or arrangement of skin nociceptors and may consequently lead to the delayed detection of liminal noxious events among strength athletes [22,63,64,65]. If we consider this strand of research, in our study, the lower sensitivity to pain recorded in the group of powerlifters, both at baseline but significantly at post-training in comparison with runners and controls, could also be associated with the influence of the different body structure detected by a higher body mass index among the anthropometric measures of the participants.

In addition to this argument, there is also one that traces the EIH of acute dynamic resistance exercise athletes to cardiovascular activity, namely the increase in BP. It is documented that those who have elevated resting BP show greater BP responses to the cold pressor test (CPT) [66]. The inverse relationship between BP and laboratory pain sensitivity has been observed with acute elevation in BP induced by pharmacological and behavioral interventions [67]. There is some evidence that those who habitually engage in high-intensity resistance exercise (e.g., weightlifters, powerlifters) exhibit higher resting BP compared to healthy controls [68,69]. In the recent study by Umeda and Okifuji [49], it was reported that resistance exercise-trained athletes did not show greater conditioned pain modulation (CPM) compared to healthy controls, albeit their greater systolic BP responses to Cold Pressor Test in comparison to healthy controls. The authors, therefore, reported that resistance exercise-trained athletes do not show a superior function of central pain inhibitory processing compared to healthy controls, despite the hypothesized physiological advantage of increased systolic BP responses on pain processing. Other studies provide mixed data about the relationship between the magnitude of BP elevations and changes in pain sensitivity [10,70].

In our study, in fact, in the comparative measurement of physiological variables (systolic and diastolic BP, HR, body temperature) collected both before and after training and both before and after CPT, it was found that the group of powerlifters reported significantly higher values of BP and HR. This difference was present at baseline but with values remaining high for powerlifters even after training, and before and after CPT, despite a slight hypotensive effect recorded in groups. Because body temperature measurements showed no significant changes and differences both before and after training and CPT and in the comparison between the groups, a possible hypothesis of specific incidence of thermoregulatory response in the three groups was not supported. Therefore, we believe that the most plausible explanatory hypothesis for the difference in perceived pain intensity between the groups should be traced back to the incidence of the pressor component, as suggested by Makovac et al. [71]. Their recent systematic meta-analysis is the first performed with reference to the distinctive effects of elevated BP on the nociceptive and perceptual components of the pain response. The results of this meta-analysis confirmed the existence of a significant association between elevated BP and hypoalgesia, and they pointed toward the need for a better understanding of its underlying mechanisms.

The systematic review by Andrade et al. [72] illustrated the positive effect of strength training treatments on patients with fibromyalgia. The 22 studies considered in the review, covering a time frame from 2001 to 2017, showed that the intervention has favorable results, such as reducing physical and psychological symptoms, and in particular, pain was significantly reduced by the strength training intervention. Among the studies conducted with fibromyalgia patients, in those by Kayo et al. [73] and Hooten et al. [74], the effects of strength training were also compared with those of aerobic exercise. It was observed that both interventions presented similar results, particularly in reducing the pain of patients.

To the best of our knowledge, no comparative studies have so far been presented to identify in (non-patient) athletes the effect of reducing the intensity of perceived pain through the administration of strength training and aerobic exercise. Therefore, in our study, the comparison of powerlifters with endurance runners constitutes a pilot study to better investigate the effect on pain modulation processes of the constant maximal explosive force training that characterizes the athletic profile of the powerlifter. This represents a step forward with respect to previous studies which, with regard to the hypoalgesic effect of anaerobic exercise, mainly considered dynamic resistance exercises, which involve a predominantly moderate intensity workload (60–75% 1 RM), whereas powerlifter training rather involves predominantly high-intensity work (>75% 1 RM).

The differences in our study at baseline, but very significantly after the strength activation session in the powerlifters, provide interesting insights into the hypoalgesic effect of high-intensity strength training. With regard to the limited hypoalgesic response reported by the endurance runners after the treadmill session, one possible explanation could be due to the duration of the aerobic activation session. It is likely that forty minutes was not sufficient for them to adequately activate the modulatory inhibition of pain, as they are used to competing with runs of much longer duration; whereas in powerlifters, the explosive strength training session through the intense pyramid loading program interspersed with four-minute recoveries was shown to be capable of activating a vigorous hypoalgesic response. Additional studies will definitely be required in order to find further evidence for these findings and to better control for experience and environmental variables, setting, or other factors that may have an influence on the specific appraisal of perceived pain.

This work must clearly be considered in relation to several limitations. First of all, among the anthropometric characteristics that might have shown an impact on the results, the measure of various proportions of subcutaneous fat tissue between groups of athletes was not considered. This difference could actually affect the thermal conductivity. A further limitation of the work is that it involved only male athletes, and therefore a replication with a female sample would be essential in order to obtain data on analgesic response also in relation to gender.

The results must also be evaluated in light of the short time between tests. The two-minute time limit was imposed by a precautionary decision aimed at ensuring the utmost safety of the study’s participants while also providing a period of time deemed useful for stimulating the response, as indicated by Peckerman et al. [75], who suggest that 90–120 s of stimulation should be sufficient to obtain a true peak response. In any case, the extension of the CPT to three minutes, carried out in other previous studies [76,77,78], could possibly have affected the process of inhibitory control and modulation of perceived pain in a different way within the endurance runners. Furthermore, the comparative evaluation of the intensity of pain perceived by the two groups of athletes and controls for several time points, and not just a single detection at the end of the 120 s CPT test, could indicate from which time point on groups differ after training.

A further limitation is attributable to the fact that the circadian effect in athletes was not evaluated in the study, and all trials were conducted in the morning interval (9 a.m.–12 p.m.). A continuation of the research should also contemplate the evaluation of the circadian effect on athletes’ performance. Monitoring participants’ hydration could also have been another measure worthy of attention, as acute hypohydration may reduce endothelial function, alter BP regulation and increase sympathetic nervous system activity, and worsen orthostatic tolerance.

In addition, a limitation of the work is not having in-depth statistical analysis for BP as a magnitude-based inference, mixed effect modeling technique with the control of covariates. Furthermore, a 24 h BP Monitoring of mean arterial pressure (MAP), double product (DP), and pulse rate (PR) could have been a useful measure to assess an acute response to the exercise sessions. Recently, it was reported that these statistical analyses regarding BP responsiveness could improve interpretation and achieve higher reliability for future studies in exercise science [79].

Furthermore, the small number of participants could reduce the generalizability of the results. The evaluation of the effect of strength training could be further investigated by setting up another comparison between powerlifters and bodybuilders, taking into account their different approaches to athletic work in terms of load, intensity, and structuring of strength training sequences. The results could provide useful insights into the relationships between the load and structuring of anaerobic work and the corresponding measure of analgesic effect.

An extension of the study could consider the influence of covariates such as anthropometric characteristics, age, competitive experience, intensity and frequency of training, number of possible injuries, psychological attitude towards pain (fear of pain, catastrophization), and coping styles.

## 5. Conclusions

The study has shown, firstly, that the practice of aerobic training in runners and explosive strength training in powerlifters determines, even at a baseline level, a lower perception of pain compared with non-athlete controls; secondly, these differences can be amplified after the administration of a single training session that stimulates aerobic and anaerobic activation in the two groups, respectively. When compared to the group of endurance runners and controls, the results showed that the decrease in perceived pain response in the CPT test is particularly robust in powerlifters following strength training. Since the comparative measurement of physiological variables showed that the group of powerlifters, compared to runners and controls, reported significantly higher values of BP both before and after training and CPT, the most plausible explanatory hypothesis of the difference in intensity of perceived pain between the two groups of athletes traces back to the incidence of the pressure component. Future research should follow this by expanding methods and analyses related to blood pressor responsiveness. Results suggest further extensions of the study to investigate the underlying mechanisms between high BP and hypoalgesia through the induction of strength training.

## Figures and Tables

**Table 1:** Baseline anthropometric data of participants.

	Endurance Runners (n = 22) (Mean ± SD)	Powerlifters (n = 22) (Mean ± SD)	Controls (n = 22) (Mean ± SD)	*p*-Value
Age (years)	22.36 ± 2.59	23.09 ± 4.83	22.70 ± 3.68	0.82
Height (cm)	175.57 ± 2.78	178.21 ± 3.72	169.03 ± 3.24	<0.001
Weight (kg)	64.22 ± 1.75	80.32 ± 9.81	71.34 ± 6.20	<0.001
Body mass index (kg/m^2^)	20.82 ± 0.31	25.29 ± 4.08	24.05 ± 1.22	<0.001
Training hours (h*week)	10.21 ± 2.30	11.42 ± 2.80		<0.01
Sessions (sessions per week)	3.38 ± 0.77	4.93 ± 1.41		<0.001
Training experience (years)	6.18 ± 4.29	5.23 ± 6.92		0.59

Data is presented as the mean (SD). Training hours: self-reported hours of weekly training/physical exercise; Sessions: number of self-reported training/physical exercise sessions per week.

**Table 2:** Physiological values in participants before and immediately after CPT (Baseline).

	Powerlifters						Runners						Controls					
	*PRE* CPT			*POST* CPT			*PRE* CPT			*POST* CPT			*PRE* CPT			*POST* CPT		
	DBP	SBP	HR	DBP	SBP	HR	DBP	SBP	HR	DBP	SBP	HR	DBP	SBP	HR	DBP	SBP	HR
			HR	DBP	SBP	HR	DBP	SBP	HR	DBP	SBP	HR	DBP	SBP		DBP	SBP	HR
M	80.5	138.8	82.7	82.7	141.9	81.5	73.4	123.9	67.0	74.4	120.9	69.4	74.6	119.4	68.5	90.3	137.7	69.7
SD	±3.5	±6.9	±3.6	±3.6	±6.3	±3.5	±3.7	±4.6	±4.6	±2.5	±4.6	±4.8	±2.4	±2.1	±1.8	±3.2	±3.7	±2.6

CPT: Cold Pressor Test; DBP (mmHg): Diastolic Blood Pressure; SBP (mmHg): Systolic Blood Pressure; HR (bpm): Heart Rate (beat per minute); M: Mean; SD: Standard Deviation.

**Table 3:** Significant differences between physiological measurements collected in the three groups.

		Powerlifters (n = 22) (Mean ± SD)	Endurance Runners (n = 22) (Mean ± SD)	Controls (n = 22) (Mean ± SD)	F	*p*-Value	ηp^2^
	DBP (mmHg)	79.73 ± 3.83	72.23 ± 3.02	74.90 ± 4.19	22.892	<0.001	0.425
*PRE* Aerobic Training	SBP (mmHg)	142.27 ± 4.47	119.55 ± 5.04	124.90 ± 8.80	76.698	<0.001	0.712
	HR (bpm)	76.23 ± 3.77	63.27 ± 5.09	69.90 ± 3.66	51.563	<0.001	0.625
	Body temp. (°C)	36.06 ± 0.42	36.13 ± 0.25	36.23 ± 0.25	1.826	>0.05	0.056
	DBP (mmHg)	78.00 ± 3.88	66.32 ± 2.88	73.14 ± 4.36	53.895	<0.001	0.635
*POST* Aerobic Training	SBP (mmHg)	140.59 ± 4.81	114.68 ± 4.05	123.24 ± 7.75	116.852	<0.001	0.790
	HR (bpm)	110.68 ± 5.51	93.27 ± 5.82	103.86 ± 10.34	30.135	<0.001	0.493
	Body temp. (°C)	36.58 ± 0.18	36.59 ± 0.19	36.61 ± 0.18	0.176	>0.05	0.006
	DBP (mmHg)	79.18 ± 4.11	68.32 ± 2.99	74.23 ± 4.12	45.559	<0.001	0.591
*PRE* CPT	SBP (mmHg)	141.64 ± 3.57	115.86 ± 3.83	124.82 ± 6.81	153.199	<0.001	0.829
	HR (bpm)	99.00 ± 6.56	88.73 ± 6.45	92.32 ± 7.93	13.221	<0.001	0.296
	Body temp. (°C)	36.44 ± 0.14	36.48 ± 0.18	36.53 ± 0.16	1.803	>0.05	0.053
	DBP (mmHg)	79.05 ± 4.12	69.73 ± 2.64	74.23 ± 3.88	36.641	<0.001	0.538
*POST* CPT	SBP (mmHg)	140.73 ± 3.86	113.64 ± 4.51	124.05 ± 7.58	132.987	<0.001	0.808
	HR (bpm)	89.55 ± 4.97	89.09 ± 4.76	85.95 ± 6.86	2.670	>0.05	0.078
	Body temp. (°C)	36.43 ± 0.15	36.44 ± 0.19	36.46 ± 0.12	0198	>0.05	0.006
	DBP (mmHg)	80.27 ± 4.04	71.68 ± 3.14	76.68 ± 3.26	33.427	<0.001	0.515
*PRE* Strength Training	SBP (mmHg)	140.91 ± 4.44	119.50 ± 5.25	126.36 ± 7.59	75.206	<0.001	0.705
	HR (bpm)	77.05 ± 2.64	65.95 ± 4.55	70.73 ± 4.04	48.413	<0.001	0.596
	Body temp. (°C)	36.09 ± 0.25	36.22 ± 0.27	36.24 ± 0.31	1.949	>0.05	0.058
	DBP (mmHg)	83.77 ± 4.91	68.77 ± 3.25	74.55 ± 3.58	79.565	<0.001	0.716
*POST* Strength Training	SBP (mmHg)	139.95 ± 4.05	116.41 ± 5.06	123.77 ± 7.82	92.827	<0.001	0.747
	HR (bpm)	105.18 ± 4.72	77.95 ± 6.36	95.82 ± 9.26	85.073	<0.001	0.730
	Body temp. (°C)	36.59 ± 0.22	36.51 ± 0.23	36.50 ± 0.24	0.939	>0.05	0.029
	DBP (mmHg)	80.09 ± 5.19	70.23 ± 3.32	76.14 ± 3.55	32.151	<0.001	0.505
*PRE* CPT	SBP (mmHg)	137.05 ± 3.47	117.91 ± 4.86	126.32 ± 8.29	58.182	<0.001	0.649
	HR (bpm)	94.14 ± 4.39	72.73 ± 5.42	84.95 ± 7.13	76.579	<0.001	0.709
	Body temp. (°C)	36.44 ± 0.24	36.41 ± 0.18	36.42 ± 0.18	0.182	>0.05	0.006
	DBP (mmHg)	82.68 ± 4.71	71.73 ± 2.64	76.77 ± 3.68	44.451	<0.001	0.596
*POST* CPT	SBP (mmHg)	133.86 ± 2.90	119.64 ± 5.02	127.82 ± 7.77	35.816	<0.001	0.532
	HR (bpm)	86.18 ± 6.28	69.32 ± 4.93	76.32 ± 5.92	47.857	>0.05	0.603
	Body temp. (°C)	36.39 ± 0.21	36.34 ± 0.13	36.33 ± 0.16	0.966	>0.05	0.030

Data is presented as the mean (SD). DBP (mmHg): Diastolic Blood Pressure; SBP (mmHg): Systolic Blood Pressure; HR (bpm): Heart Rate (beat per minute); Body temp. (°C): Body Temperature (Celsius degrees); ηp^2^: partial eta-squared.

## Data Availability

The datasets during and/or analyzed during the current study are available from the corresponding author on reasonable request.

## Supplementary Materials

The following supporting information can be downloaded at: https://www.mdpi.com/article/10.3390/sports10120211/s1, Table S1: Physiological values in Powerlifters before and after Strength Training-before and after CPT; Table S2: Physiological values in Powerlifters before and after Aerobic Training-before and after CPT; Table S3: Physiological values in Runners before and after Aerobic Training-before and after CPT; Table S4: Physiological values in Runners before and after Strength Training-before and after CPT; Table S5: Physiological values in Controls before and after Aerobic Training-before and after CPT; Table S6: Physiological values in Controls before and after Strength Training-before and after CPT.
