# Supplementary material for: Perceived Pain in Athletes: A Comparison between Endurance Runners and Powerlifters through a Cold Experimental Stimulation and Two Sessions of Various Physical Activation
Source: Sports (Basel). 2022 Dec 19;10(12):211. doi: 10.3390/sports10120211 (PMC9785022; doi:10.3390/sports10120211)
Supplement: Supplementary file 1 [file sports-10-00211-s001.zip › sports-1824204-supplementary.pdf]

Table S1 Physiological values in Powerlifters before and after Strength Training - before and after CPT

Powerlifters – Strength Training															
***PRE* training**				***POST* training**				***PRE* CPT**				***POST* CPT**			
DBP	SBP	HR	TEMP	DBP	SBP	HR	TEMP	DBP	SBP	HR	TEMP	DBP	SBP	HR	TEMP
85	148	80	35.9	92	146	108	36.4	86	141	98	36.2	88	138	93	36.1
88	141	76	36.1	92	139	106	36.9	88	136	94	36.7	89	135	86	36.4
78	145	78	36.2	81	143	111	36.7	74	137	100	36.6	78	135	96	36.4
76	139	76	36.0	75	137	106	36.5	77	135	98	36.3	78	131	94	36.3
87	148	78	36.1	84	147	108	36.8	78	142	96	36.6	81	136	97	36.5
76	140	80	35.8	80	138	113	36.4	77	134	102	36.4	80	132	94	36.3
80	138	78	36.3	76	142	107	36.9	73	137	94	36.8	78	134	86	36.7
79	145	82	36.3	83	142	110	36.7	75	136	98	36.7	79	134	93	36.6
79	142	76	36.4	84	139	98	36.6	82	135	87	36.5	84	130	79	36.6
80	136	75	36.2	86	138	101	36.8	80	138	91	36.7	83	138	85	36.7
82	138	76	35.8	82	136	102	36.5	79	134	94	36.3	84	136	85	36.2
77	137	72	35.7	82	135	98	36.3	76	130	86	36.2	77	128	79	36.3
84	143	81	36.0	91	146	105	36.7	89	141	96	36.5	91	136	90	36.5
76	140	75	36.4	82	137	98	36.6	82	140	96	36.3	80	135	85	36.3
85	149	74	36.1	90	146	104	36.3	88	142	95	36.2	91	134	87	36.3
80	141	78	36.5	78	143	108	36.9	75	136	92	36.7	79	132	80	36.6
74	138	76	35.8	82	136	106	36.4	84	136	95	36.4	85	132	82	36.4
82	142	76	36.2	88	138	100	36.5	86	141	88	36.3	87	130	76	36.2
81	144	80	36.3	85	142	112	36.7	79	137	96	36.6	81	140	88	36.5
76	134	72	35.9	81	132	98	36.3	75	130	86	36.1	81	132	78	36.1
85	132	78	35.6	89	138	107	36.2	85	136	97	35.9	89	133	83	36.0
76	140	78	36.4	80	139	108	36.8	74	141	92	36.7	76	134	80	36.7

CPT: Cold Pressor Test; DBP (mmHg): Diastolic Blood Pressure; SBP (mmHg): Systolic Blood Pressure; HR (bpm): Heart Rate (beat per minute); Temp: Body Temperature (Celsius degrees).

Table S2 Physiological values in Powerlifters before and after Aerobic Training - before and after CPT

**Powerlifters - Aerobic Training**															
***PRE* training**				***POST* training**				***PRE* CPT**				***POST* CPT**			
DBP	SBP	HR	Temp	DBP	SBP	HR	Temp	DBP	SBP	HR	Temp	DBP	SBP	HR	Temp
80	146	76	35.8	78	147	110	36.7	79	145	99	36.5	80	144	94	36.6
87	143	80	36.5	85	145	109	36.6	88	144	96	36.6	86	146	89	36.6
78	144	75	36.0	76	138	107	36.7	76	142	94	36.4	78	142	85	36.3
77	142	81	36.4	78	140	110	36.4	79	142	102	36.3	81	141	98	36.2
87	148	84	35.8	84	136	116	36.5	86	138	104	36.2	86	140	89	36.3
78	145	76	36.4	80	144	118	36.7	79	143	106	36.5	80	141	93	36.4
82	140	79	36.2	80	140	112	36.8	81	141	102	36.7	81	138	99	36.5
76	138	79	35.9	73	142	110	36.7	74	139	98	36.5	76	136	88	36.5
83	146	80	36.6	78	136	99	36.6	81	140	86	36.4	82	139	87	36.6
80	138	73	35.9	74	132	115	36.5	75	134	103	36.3	75	134	86	36.4
81	136	75	36.2	79	142	112	36.3	78	144	101	36.2	79	146	90	36.5
78	143	72	35.8	77	141	109	36.8	78	144	98	36.4	75	140	93	36.3
80	138	70	35.4	83	132	116	36.6	83	139	106	36.3	81	138	85	36.2
76	141	74	36.6	76	146	108	36.4	78	144	98	36.5	82	142	89	36.5
85	149	73	35.6	82	151	107	36.4	83	150	92	36.3	78	148	83	36.4
75	134	76	36.2	72	138	120	36.6	73	136	112	36.6	70	136	94	36.4
76	146	78	36.3	74	136	116	36.8	84	138	101	36.6	84	136	98	36.6
80	140	74	35.6	76	138	115	36.7	78	140	106	36.3	80	138	89	36.2
83	148	82	36.5	81	145	112	36.8	80	144	99	36.5	78	146	86	36.5
74	138	70	36.3	73	142	100	36.3	73	141	88	36.4	73	138	81	36.3
83	149	74	36.4	83	144	102	36.7	82	146	87	36.6	81	144	84	36.5
75	138	76	35.0	74	138	112	36.2	74	142	100	36.5	73	143	90	36.7

CPT: Cold Pressor Test; DBP (mmHg): Diastolic Blood Pressure; SBP (mmHg): Systolic Blood Pressure; HR (bpm): Heart Rate (beat per minute); Temp: Body Temperature (Celsius degrees).

Table S3 Physiological values in Runners before and after Aerobic Training - before and after CPT

**Runners – Aerobic Training**															
***PRE* training**				***POST* training**				***PRE* CPT**				***POST* CPT**			
DBP	SBP	HR	Temp	DBP	SBP	HR	Temp	DBP	SBP	HR	Temp	DBP	SBP	HR	Temp
75	108	55	36.1	68	106	85	36.6	70	111	80	36.5	72	110	85	36.5
74	118	65	36.5	71	114	90	36.7	73	112	87	36.7	74	109	88	36.6
72	126	60	36.4	68	120	98	36.8	70	122	92	36.5	72	119	90	36.6
73	113	62	36.0	66	112	89	36.4	68	110	85	36.2	69	108	87	36.1
70	115	58	35.8	64	109	84	36.3	68	112	83	36.3	71	110	85	36.3
67	116	58	35.9	62	112	86	36.5	66	114	80	36.4	70	112	82	36.4
75	120	68	36.2	64	118	91	36.6	65	117	89	36.5	64	115	90	36.3
64	115	71	36.0	62	111	85	36.4	62	114	78	36.4	64	112	76	36.5
70	115	58	36.3	68	112	86	36.8	67	110	83	36.7	69	108	85	36.7
76	121	65	35.9	69	115	93	36.7	70	115	88	36.5	72	118	86	36.4
75	113	62	36.2	68	108	96	36.7	71	112	92	36.6	72	110	89	36.6
76	123	72	36.4	72	115	102	36.6	72	120	94	36.3	70	118	96	36.4
71	120	58	35.8	65	116	92	36.5	64	113	90	36.5	66	107	94	36.4
73	126	63	36.2	66	118	96	36.9	68	120	92	36.8	71	116	88	36.8
72	118	62	35.9	66	112	97	36.3	67	116	94	36.2	69	110	96	36.3
73	120	57	36.1	67	116	91	36.7	72	119	86	36.5	71	116	90	36.4
71	123	60	36.5	65	118	96	36.8	65	116	94	36.7	68	121	92	36.6
75	126	71	36.3	71	120	104	36.5	72	119	98	36.5	71	116	92	36.4
73	126	66	35.8	64	117	99	36.4	66	121	95	36.2	68	116	94	36.2
71	125	68	36.1	63	121	98	36.6	68	119	93	36.5	70	122	92	36.3
68	120	63	35.9	63	115	95	36.3	67	116	91	36.3	68	110	93	36.1
75	123	70	36.6	67	118	99	36.9	72	121	88	36.7	73	117	90	36.7

CPT: Cold Pressor Test; DBP (mmHg): Diastolic Blood Pressure; SBP (mmHg): Systolic Blood Pressure; HR (bpm): Heart Rate (beat per minute); Temp: Body Temperature (Celsius degrees).

Table S4 Physiological values in Runners before and after Strength Training - before and after CPT

Runners – Strength Training															
***PRE* training**				***POST* training**				***PRE* CPT**				***POST* CPT**			
DBP	SBP	HR	Temp	DBP	SBP	HR	Temp	DBP	SBP	HR	Temp	DBP	SBP	HR	Temp
78	110	64	36.2	76	109	78	36.7	76	110	74	36.6	77	110	72	36.6
73	120	66	36.5	68	118	76	36.6	70	120	78	36.6	72	121	74	36.5
71	124	59	36.2	66	121	74	36.7	68	124	68	36.5	71	123	67	36.4
73	116	65	36.1	68	112	73	36.4	70	115	70	36.3	71	118	68	36.3
68	110	60	35.4	66	108	71	35.8	71	112	68	35.9	73	114	65	36.0
67	116	62	36.1	65	113	73	36.3	69	118	74	36.5	70	118	69	36.4
71	118	66	36.1	68	120	78	36.3	71	116	73	36.2	72	120	67	36.3
68	120	68	36.4	70	122	85	36.6	69	119	78	36.2	69	121	75	36.2
72	121	62	36.2	70	117	71	36.6	71	120	68	36.5	72	119	70	36.5
73	115	70	36.4	69	112	81	36.7	71	114	78	36.4	74	118	70	36.5
72	116	71	36.6	68	113	84	36.8	68	112	81	36.6	71	113	75	36.4
74	119	71	36.4	72	115	83	36.6	73	118	74	36.4	73	120	71	36.3
72	118	64	35.8	67	110	76	36.2	69	117	72	36.4	70	118	66	36.4
66	115	65	36.6	64	116	77	36.8	65	113	67	36.5	68	117	67	36.4
72	118	66	36.1	69	111	75	36.4	70	112	67	36.4	72	114	62	36.2
74	122	62	36.3	74	121	73	36.6	76	119	70	36.4	74	118	67	36.3
70	120	59	36.2	68	116	68	36.4	64	117	63	36.3	67	124	59	36.2
75	128	68	36.2	73	119	76	36.6	75	121	73	36.7	75	118	69	36.3
71	125	64	36.0	67	122	78	36.3	69	125	66	36.4	71	128	65	36.4
68	120	70	36.3	66	116	81	36.6	65	118	75	36.2	67	121	72	36.2
71	128	73	36.2	65	124	89	36.5	70	126	79	36.4	73	128	73	36.3
78	130	76	36.6	74	126	95	36.7	75	128	84	36.5	76	131	82	36.3

CPT: Cold Pressor Test; DBP (mmHg): Diastolic Blood Pressure; SBP (mmHg): Systolic Blood Pressure; HR (bpm): Heart Rate (beat per minute); Temp: Body Temperature (Celsius degrees).

Table S5 Physiological values in Controls before and after Aerobic Training - before and after CPT

Controls – Aerobic Training															
***PRE* training**				***POST* training**				***PRE* CPT**				***POST* CPT**			
DBP	SBP	HR	Temp	DBP	SBP	HR	Temp	DBP	SBP	HR	Temp	DBP	SBP	HR	Temp
72	122	73	36.3	68	123	110	36.6	71	124	106	36.6	72	123	98	36.6
80	136	73	36.4	78	132	114	36.5	77	134	109	36.5	76	133	100	36.4
78	141	68	36.2	76	139	89	36.6	78	136	85	36.5	78	135	80	36.5
72	118	65	36.3	73	117	90	36.8	74	118	84	36.7	72	106	79	36.5
80	120	69	36.1	76	118	111	36.4	80	120	96	36.5	79	121	89	36.6
75	122	72	36.1	77	125	96	36.7	76	128	89	36.4	75	126	83	36.4
73	135	71	36.4	68	129	112	36.6	70	131	97	36.5	69	130	91	36.4
68	115	70	36.3	68	112	109	36.7	70	116	95	36.6	71	118	88	36.3
80	136	72	36.2	76	132	108	36.9	77	129	98	36.8	78	130	88	36.7
71	121	65	36.1	69	119	87	36.5	72	123	81	36.5	72	123	78	36.5
73	130	74	36.2	69	128	113	36.7	72	130	94	36.6	74	129	90	36.4
72	116	71	36.2	73	118	109	36.5	74	122	96	36.4	73	121	91	36.4
84	142	73	36.3	82	138	115	36.8	84	140	98	36.7	82	139	92	36.6
72	123	70	36.1	68	121	106	37.0	67	119	92	36.8	68	120	87	36.6
75	119	67	36.1	76	120	94	36.7	78	123	88	36.6	80	125	83	36.5
74	118	76	36.4	67	115	112	36.6	71	120	90	36.6	70	119	85	36.4
80	132	75	36.4	78	126	108	36.6	76	130	96	36.5	76	130	86	36.5
76	124	70	36.3	78	123	116	36.5	78	120	98	36.5	79	123	87	36.4
68	120	66	36.0	71	124	106	36.2	68	123	96	36.1	70	120	90	36.2
76	112	65	36.3	73	110	89	36.5	72	112	82	36.3	71	110	76	36.4
74	121	63	36.2	72	119	87	36.4	74	125	80	36.4	73	122	76	36.3
76	124	69	36.3	73	122	92	36.7	74	123	81	36.5	75	126	74	36.5

CPT: Cold Pressor Test; DBP (mmHg): Diastolic Blood Pressure; SBP (mmHg): Systolic Blood Pressure; HR (bpm): Heart Rate (beat per minute); Temp: Body Temperature (Celsius degrees).

Table S6 Physiological values in Controls before and after Strength Training - before and after CPT

**Controls – Strength Training**															
***PRE* training**				***POST* training**				***PRE* CPT**				***POST* CPT**			
DBP	SBP	HR	Temp	DBP	SBP	HR	Temp	DBP	SBP	HR	Temp	DBP	SBP	HR	Temp
75	118	68	36.2	71	116	80	36.4	72	117	78	36.4	71	116	76	36.4
80	128	73	36.3	79	126	91	36.6	80	128	87	36.7	79	128	79	36.6
76	122	67	36.1	74	119	89	36.4	76	121	83	36.4	75	119	80	36.4
76	124	70	36.5	73	120	85	36.6	78	123	80	36.4	80	125	73	36.3
81	126	65	35.4	78	122	83	36.0	80	124	77	36.2	80	122	70	36.1
78	118	66	36.2	77	115	80	36.4	80	120	72	36.3	81	122	68	36.2
83	135	80	36.6	82	136	106	36.7	81	138	92	36.7	82	140	85	36.5
74	125	76	36.1	76	123	99	36.3	78	128	82	36.2	78	130	77	36.2
72	121	71	36.3	70	119	102	36.6	72	121	91	36.4	71	120	81	36.3
75	120	69	36.7	73	118	95	36.9	74	119	88	36.8	76	125	78	36.1
74	130	68	36.2	72	129	98	36.4	71	132	80	36.4	72	136	73	36.3
80	139	75	36.6	78	136	105	36.8	80	140	96	36.6	81	142	84	36.7
82	140	77	36.2	79	138	110	36.5	78	141	98	36.4	80	140	88	36.3
74	120	72	36.1	71	117	100	36.4	74	119	83	36.3	77	128	71	36.3
75	118	75	36.4	73	116	112	36.6	74	118	97	36.4	76	127	82	36.5
76	124	68	36.0	73	120	90	36.3	72	121	84	36.3	72	119	72	36.2
79	135	70	36.7	77	131	99	36.9	81	137	83	36.7	79	135	74	36.5
76	115	72	36.2	73	112	101	36.6	75	114	92	36.5	75	118	83	36.5
78	127	65	35.8	77	124	94	36.1	78	124	79	36.1	80	127	68	36.3
79	141	72	36.5	76	138	106	36.7	78	140	89	36.4	76	138	76	36.2
73	126	67	35.9	70	123	89	36.2	73	126	77	36.2	78	129	67	36.2
71	128	70	36.4	68	125	94	36.6	70	128	81	36.4	70	126	74	36.2

CPT: Cold Pressor Test; DBP (mmHg): Diastolic Blood Pressure; SBP (mmHg): Systolic Blood Pressure; HR (bpm): Heart Rate (beat per minute); Temp: Body Temperature (Celsius degrees).
